# Client and family experiences with telehealth‐delivered early psychosis services

**DOI:** 10.1111/eip.13550

**Published:** 2024-06-30

**Authors:** Ana Carolina Florence, Ana Stefančić, Adrienne Sheitman, Kaleigh Fidaleo, Iruma Bello, Lisa Dixon, Robert Eldon Drake, Ilana Nossel, Leopoldo J. Cabassa, Elaina Montague, Shannon Pagdon, Jamaitreya Lyn, Sapana R. Patel

**Affiliations:** ^1^ New York State Psychiatric Institute New York New York USA; ^2^ Department of Psychiatry Columbia University New York New York USA; ^3^ Division of Clinical Therapeutics New York State Psychiatric Institute New York New York USA; ^4^ Columbia University Vagelos College of Physicians and Surgeons New York New York USA; ^5^ Columbia University Irving Medical Center New York New York USA; ^6^ George Warren Brown School of Social Work, Washington University in St. Louis St Louis Missouri USA; ^7^ Zucker Hillside Hospital New York New York USA; ^8^ University of Pittsburgh Pittsburgh Pennsylvania USA; ^9^ New York USA

**Keywords:** COVID‐19 pandemic, early intervention for psychosis, family, mental health services, qualitative research, telehealth, youth

## Abstract

**Objective:**

The COVID‐19 pandemic prompted a significant shift to delivering early psychosis services using telehealth. Little is known about the experience of using telehealth in early psychosis services. This quality improvement qualitative project investigated the experiences of program participants and family members with telehealth services in OnTrackNY, an early intervention program for psychosis in New York State during the COVID‐19 pandemic.

**Methods:**

The project team conducted individual interviews and focus groups. Data analyses used a matrix approach.

**Results:**

Nineteen OnTrackNY program participants and nine family members participated in five focus groups and nine individual interviews. Data were organized into five themes (a) accessibility: most individuals had a device and internet access and challenges were related to connectivity, such as image freezing and sound breaking; (b) convenience/flexibility: benefits included the reduced commute and costs; (c) levels of comfort/privacy with telehealth: program participants felt less judged and less anxiety leading up to in‐person appointments while also expressing privacy concerns; (d) sense of connectedness: in‐person social connections were deemed important and not replaceable by telehealth; and (e) suggestions: program participants expressed a preference for in‐person group activities and suggested hybrid options, highlighting the importance of in‐person visits to establish rapport at the beginning of treatment before transitioning to telehealth.

**Conclusions:**

Telehealth services were generally well accepted. Suggestions for future service delivery include offering a combination of telehealth and in‐person services based on program participants' preferences and prioritizing in‐person services during the early phase of treatment.

## 1 INTRODUCTION

The rapid rise of COVID‐19 prompted significant changes to mental health care systems, including a rapid increase in the use of telehealth services (i.e., care delivered using technology such as videoconferencing or phone) (Zangani et al., 2022). Evidence suggests that telehealth services yield similar outcomes as in person in various clinical populations, especially in the short term and when using videoconferencing (Hubley et al., 2016; Santesteban‐Echarri et al., 2020). Studies of telehealth services during the pandemic have identified benefits such as increased access, convenience and reduced travel costs, but also some drawbacks, such as digital exclusion, technological difficulties, and privacy concerns (Abraham et al., 2021; Appleton et al., 2021; di Carlo et al., 2021; Siegel et al., 2021). Research on telehealth to date has focused more on general mental health services than early intervention in psychosis, with the use of surveys and provider perspectives predominating in the literature (Hailey, Roine, & Ohinmaa, 2008; Lipschitz et al., 2022; Nicholas et al., 2021, Doran & Lawson, 2021).

OnTrackNY, an early intervention program for psychosis in New York State, uses a team‐based multidisciplinary approach offering young people ages 16–30 and their families care management, cognitive behavioural therapy‐based psychotherapy, medication management, health and wellness interventions, family psychoeducation and support, supported employment and education services and peer support to promote recovery (Bello et al., 2017; Heinssen et al., 2014). OnTrackNY is flexible and teams work with participants through a shared decision‐making framework to decide on frequency of treatment and interventions that are aligned with young people's goals.

In early intervention programs for psychosis, staff and providers demonstrate positive attitudes towards the use of telehealth to enhance treatment, and program participants are more likely to endorse the use of telehealth than providers (Camacho & Torous, 2021; Dong et al., 2023). Initial studies investigating telehealth services for early psychosis during COVID‐19 have reported high levels of engagement and good acceptability among program participants, despite concerns related to privacy and access to technology (Dong et al., 2023; Lal et al., 2022; Randall et al., 2022). While these findings are promising, there have been only three such studies to our knowledge, all using surveys, potentially limiting more in‐depth and nuanced understanding of program participants' perspectives. Further, despite family members being a key stakeholder group in early psychosis care, no studies to date have investigated their perspectives on telehealth use in early intervention programs for psychosis. Family involvement is an important part of early psychosis treatment and family psychoeducation has been associated with positive outcomes for young people (McFarlane et al., 2003). Families are often part of pathways to care and family interventions for psychosis reduce relapse rates and duration of hospitalizations (Camacho‐Gomez & Castellvi, 2020). Family involvement in OnTrackNY is based on participant preference and it is an indicator in fidelity assessments. The majority of OnTrackNY participants (94%) select some type of family involvement at enrolment and only 6% refuse it altogether (Jones et al., 2021).

Telehealth will likely continue to be a modality of care in early intervention programs for psychosis. Understanding the benefits, challenges and characterizing the experiences of program participants and their family members is crucial to maintain the standard of care and take advantage of this modality. As a hub of the Early Psychosis Intervention Network learning healthcare system (Humensky et al., 2020), we conducted a quality improvement (QI) project to support program planning and engage stakeholders to qualitatively explore program participant and family experiences with telehealth services in OnTrackNY, including benefits, challenges and suggestions for future delivery.

## 2 METHODS

### 2.1 Project overview

**FIGURE 1:**
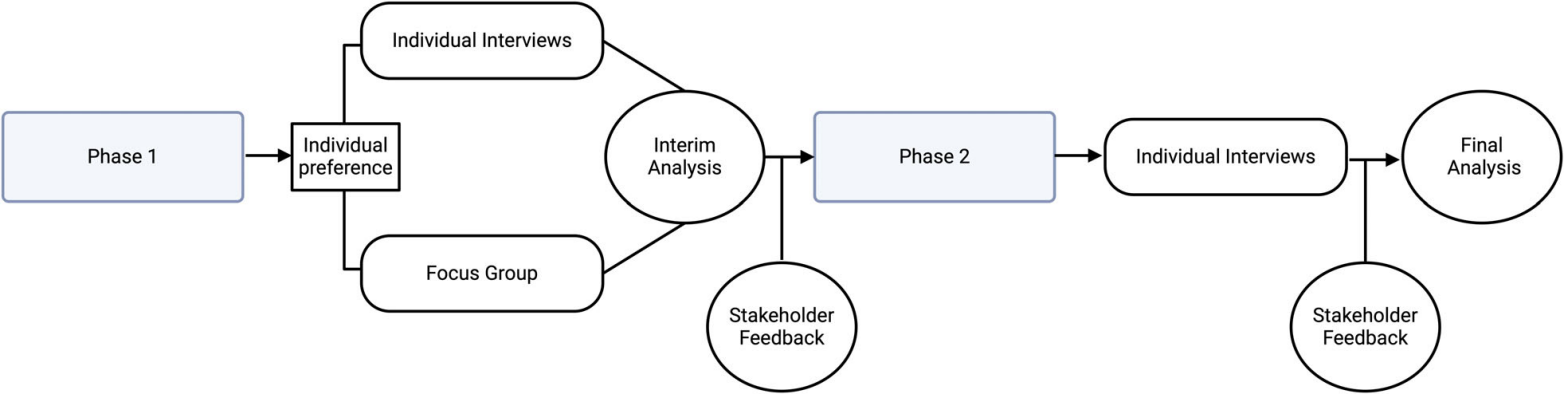
Project design.

We used a combination of interviews and focus groups to ascertain program participant and family member perspectives on telehealth services in two phases. Phase 1 explored the impact of COVID‐19 on OnTrackNY program participants (hereafter participants) and families in several life areas (e.g., social relationships, education/employment, health), and their experiences with OnTrackNY services using telehealth. Interim analysis and stakeholder feedback of Phase 1 QI data identified few challenges with the use of telehealth from participants' perspectives. Thus, Phase 2 shifted recruitment strategies and focused on soliciting information specifically about participants' challenges with telehealth. An in‐depth description of the project's partnership with individuals with lived experience, methods, and results regarding the impact of COVID‐19 on life areas other than engagement with OnTrackNY are presented elsewhere (Patel et al., 2024). This project was deemed quality improvement by the New York State Psychiatric Institute Institutional Review Board. Figure 1 depicts the project design.

### 2.2 Sample

Phase 1 used a convenience sampling approach to recruit currently or formerly enrolled OnTrackNY participants and their family members across the 25 sites in the State of New York, who had received, or had a family member who had received, OnTrackNY telehealth services. Recruitment consisted of emails to individuals who had previously expressed interest in being contacted for OnTrackNY‐related projects. Additionally, the team delivered project presentations at meetings with the OnTrackNY Youth and Young Adult Leadership Council and the Family Advisory Council—groups that provide a space for participants, graduates and family members to provide input and guidance regarding the OnTrackNY program. In Phase 2, we used a purposive sampling approach to recruit currently or formerly enrolled OnTrackNY participants who self‐reported challenges using OnTrackNY telehealth services to the project team.

### 2.3 Procedures

During both phases, interview and focus group guides were collaboratively developed by individuals with personal and/or family lived experience. These guides received feedback from researchers with qualitative expertise. Phase 1 interview guides comprised questions about experiences with OnTrackNY telehealth services with additional in‐depth probes regarding potential telehealth challenges. Family interview guides included questions about their own experiences receiving OnTrackNY services and their experiences with their loved one's care in OnTrackNY. Phase 2 interview guides focused on challenges experienced with telehealth. Interviewers met with participants to confirm their status as a current of former program participant or family member. The team then obtained verbal consent to participate in interviews or focus groups and audio recordings. Team members with personal and/or family lived experience facilitated interviews and focus groups. Focus groups and interviews were audio‐recorded, professionally transcribed, de‐identified and reviewed for accuracy. In both phases, participants, and family members self‐reported demographic information through an online Qualtrics survey.

### 2.4 Data analysis

The team used an interview summary and matrix analysis approach to analyse the QI data, described in detail elsewhere (Patel et al., 2024). Interviewers/focus group facilitators completed summary templates structured by topic (e.g., impact of pandemic, telehealth experiences) after each data collection activity. The team developed a preliminary case matrix consisting of rows for each participant or family member's data and broad descriptive column headings categorizing data (e.g., telehealth flexibility/convenience). Project team members, including individuals with lived experience, extracted data into the matrix and further summarized findings using descriptive headings and including relevant quotes. Matrix headings and data groupings were revised iteratively and team members reviewed the completed matrix to identify patterns and contrasts within and across the various domains of telehealth and participant groups. Analyses focused on both identifying patterns while also reporting single or negative cases that offered a unique or contradictory perspective. Strategies to maximize rigour included progressively reducing the data through transcribing, summarizing and charting; having multiple team members extract, categorize and interpret data; and conducting frequent debrief meetings (Creswell & Creswell, 2017; Padgett, 2011). We also sought stakeholder feedback to enhance trustworthiness of results. The Youth and Young Adult Leadership Council provided feedback on interim Phase 1 findings—which underscored the need to further explore the experiences of individuals who encountered challenges to engaging with OnTrackNY via telehealth in Phase 2. Participants in Phase 2 were invited to a virtual member checking session and reported that their perspectives were represented among the findings (Birt et al., 2016).

## 3 RESULTS

### 3.1 Project sample characteristics

Thirteen OnTrackNY participants and nine family members participated in five focus groups and three individual interviews in Phase 1 and six OnTrackNY participants participated in individual interviews in Phase 2. Table 1 shows demographic characteristics. OnTrackNY current and former participants were between the ages of 18–34 with a broad range of ethnic and racial backgrounds, were predominantly male, and most reported having some college experience or a college degree. Family members were also ethnically and racially diverse and ranged in age from 25 to over 65 years old. Family members were predominantly female, most commonly mothers or sisters of OnTrackNY participants, and most had a postgraduate degree or college degree.

**TABLE 1:** OnTrackNY participant and family member demographic characteristics.

	Participants (*n* = 19)		Family members (*n* = 9)	
	*n*	%	*n*	%
Age group				
18–24	10	52.6	0	0
25–34	8	42.1	1	11.1
35–44	0	0	1	11.1
45–54	0	0	2	22.2
55–64	0	0	1	11.1
≥65	0	0	1	11.1
Missing	1	5.3	3	33.3
Gender				
Female	7	36.8	7	77.8
Male	9	47.4	0	0
Prefer to not say/Missing	3	15.8	2	22.2
Race/Ethnicity				
Asian (non‐Hispanic)	4	21.1	2	22.2
Black (non‐Hispanic)	3	15.8	3	33.3
Hispanic	4	21.1	0	0
White (non‐Hispanic)	5	26.3	2	22.2
Other (non‐Hispanic)	1	5.3	0	0
Prefer to not say/Missing^a^	2	10.5	2	22.2
Degree or certification				
High school diploma/GED	2	10.5	1	11.1
Some college	7	36.8	0	0
College degree	8	42.1	2	22.2
Post‐graduate degree	1	5.3	4	44.4
Missing^a^	1	5.3	2	22.2
Geographic region				
New York City	18	94.7	6	66.7
Western New York	0	0	1	11.1
Missing^a^	1	5.3	2	22.2
Months in OnTrackNY				
0–6 months	1	5.3	1	11.1
7–12 months	7	36.8	2	22.2
13–18 months	3	15.8	0	0
19–24 months	3	15.8	1	11.1
25–36 months	4	21.1	1	11.1
37–48 months	0	0	4	44.4
Missing^a^	1	5.3	0	0

*Note*: Percentages may not total 100% due to rounding.

^a^ Missing denotes questions individuals skipped or refused to respond.

We identified five themes: accessibility of telehealth‐based services, convenience and flexibility, level of comfort and privacy, sense of connectedness and participants' suggestions. For each theme, we describe their benefits and challenges.

### 3.2 Accessibility of telehealth‐based services

Among the services received via telehealth, participants mentioned therapy, medication management, peer specialist services, supported employment and education services and care management (e.g., virtual career fair, and other virtual and community resources).

‘I've been having all the support I could have with psychiatric, with the therapist, with the nurse, with the peer specialist, with the person that helped me with school and work. I've been taking advantage of all [that] stuff’. (Participant).

Most participants and family members found their teams were ‘helpful and supportive’ during the pandemic and continued to offer a range of services via telehealth. Participants described using various devices (e.g., tablets, phones, computers) across different settings (e.g., home, cafe) to engage with OnTrackNY team members through text, shorter and longer phone calls and video platforms, most commonly Zoom. Most participants did not report a reduction in the frequency of services, the number of providers seen or missing sessions because they were offered via telehealth when prompted about it.

#### 3.2.1 Challenges

Some participants and family members noted challenges communicating with teams via telehealth, especially in the early stages of the pandemic when teams differed in their ability to quickly transition to telehealth services.

‘We had nothing [for probably the first couple weeks]…to be fair – I don't think anybody knew what Zoom was…They just didn't know what to do’. (Family).

These challenges were generally related to internet connectivity, experiencing audio breaking up and image freezing on virtual platforms and issues with agencies' proprietary platforms that did not work well: ‘the virtual platform] was really weak…stuff was freezing. There was a lot of glitches…’ (Participant). Furthermore, practical challenges such as connecting devices like earphones and using accessibility features on video platforms like closed captions were mentioned. When receiving calls from providers, participants talked about difficulty recognizing their caller ID, which usually didn't contain a recognizable phone number leading participants to not pick up. Nevertheless, participants and family members noted that many of these technological challenges were most prominent during the beginning of the pandemic, emphasizing that issues improved or resolved as everyone became more familiar with telehealth.

In addition to technological challenges, one participant highlighted frequent and last‐minute cancellations by teams, scheduling difficulties, especially for in‐person appointments, shorter sessions and reduced provider availability: ‘… sometimes they would cancel at the last minute’ (Participant). The same participant mentioned difficulty in accessing translation services during telehealth sessions. Participants who continued to receive in‐person care noted feeling frustrated and confused with changes in where their OnTrackNY teams were physically located during the pandemic, as well as teams having ‘lost the space where we were supposed to meet’ (Participant). Finally, one family member brought up the impact of staff turnover on communication: ‘They had changed therapists and changed doctors… They tried their best. There was a little bit of lost communication…’ (Family).

### 3.3 Convenience and flexibility

Most participants brought up the ‘*convenience*’ of receiving telehealth services, and scheduling ‘*flexibility*’, noting that telehealth reduced costs and time spent commuting and allowed participants to spend more time with family or pursuing their hobbies. Participants shared the ease with which they could reach their team compared to in‐person appointments:

‘It was way easier to be early for my appointments and catch them on time, since—I just had to tap a few buttons on a screen…’ (Participant).

Participants also appreciated quick check‐ins with their teams via text or telephone when needed. This combination of convenience and teams' responsiveness led some participants to express preference for certain telehealth services, such as quick phone check‐ins to resolve issues obtaining medications. Families expressed similar views: ‘for my son actually—I think it worked better for him. Because we're kind of far from that site…’ (Family).

#### 3.3.1 Challenges

While some participants emphasized that telehealth facilitated attendance and engagement with providers during sessions, others noted that motivation to engage was lower using telehealth: ‘If you're in the comfort of your own home, you're lazy’ (Participant). They explained that it was easier to become distracted and harder to remain engaged for the duration of virtual meetings. On the other hand, some participants valued the ability to ‘*tune out*’ during telehealth sessions. Other aspects participants valued were the flexibility to be on‐ or off‐camera in videoconferencing platforms and to choose between Zoom, phone or text. One participant noted varying expectations about keeping the camera on or off, especially in group settings.

‘I personally like it a lot…It's easy to tune out at times without feeling like you're really tuning out because you can still hear the audio, but you can turn off your camera and just walk and go get a snack and it's not limiting you to that situation’. (Participant).

Some family members expressed concern with the quality of their loved one's level of engagement in telehealth services. They highlighted that despite meeting regularly with their team, their loved ones seem to struggle with concentration and fell asleep during sessions. Given these concerns, some family members reported providing more support to ensure participants remained connected and engaged with providers over telehealth:

‘I would jump in. I would sit there with him. When he wouldn't answer, I would nudge him. He'd be half asleep…his life changed when he got isolated…[The team] really did go out of their way to make sure they would contact him…’ (Family).

### 3.4 Level of comfort and privacy

Most participants reported feeling comfortable receiving OnTrackNY services via telehealth. Many reported feeling less judged or nervous, more able to fully be themselves, or experienced less ‘*paranoia*’ leading up to appointments compared to in‐person meetings:

‘I really prefer that [telehealth], I also have too much paranoia… I feel like I'm being studied based on a very setup environment… So, just to be able to be myself wherever I am, that's the thing for me’. (Participant).

Families shared similar experiences:

‘Oh, thank God they were still giving the phone calls and the FaceTime with her for her appointments with her…[she] just couldn't wait to speak to [OnTrack team member] so she can get rid of that anxiety feeling and tell [the staff] what's going on and how it's making her feel…It relaxed her more and she always waited for those calls….’ (Family).

#### 3.4.1 Challenges

Some participants struggled to feel comfortable online and two expressed a clear preference for in‐person services. One participant mentioned cyber security as a concern: ‘I just had some experiences in the past that made me doubt my cyber security’. (Participant). Participants noted that privacy was sometimes a challenge when taking calls at home: ‘[…] everyone has to pass through my room to get to the bathroom’. (Participant). Participants noted this lack of privacy resulted from both background noise during calls and the possibility of others overhearing their conversations. While family members also mentioned overhearing sessions, they emphasized the benefit of knowing their family members were talking to their OnTrackNY team:

[I] always knew [my daughter's] appointments…I know when she's on because if I happen to be home when I'm walking past her…she'll say to me, ‘Shh. I'm on a [call]…’ (Family).

### 3.5 Sense of connectedness

Some participants expressed feeling positively connected to their providers when using telehealth, noting no difference in their sense of connectedness after transitioning to telehealth: ‘it's the same because you're still talking with them. It's just a different platform’. (Participant). When describing their connections with providers, participants reported that they seemed available, actively engaged and empathic: ‘they're just really engaged with you and your family’. (Participant). Families expressed similar views, highlighting ongoing communication: ‘The team at [OnTrackNY] was very involved with [my son]. And they were in communication constantly. And they were also open for me to call’. (Family).

#### 3.5.1 Challenges

Most participants mentioned at least one challenge with feeling connected to their team when receiving telehealth services, noting that in‐person interactions and connection are different from virtual. Participants spoke about struggles with the absence of the typical non‐verbal cues that are part of in‐person interactions, noting that body language and facial expressions, important elements for building trust and connection, do not translate well virtually: ‘I thought it was harder on the phone […] Because you don't see their body language’. (Participant). One participant noted that, unlike in‐person, it is hard to know whether someone is performing other tasks on the computer during sessions. One participant noted that building trust in person before transitioning to telehealth was helpful:

‘Luckily for me I was able to build that trust and empathy…before even heading to Zoom, so I was fairly comfortable with it. But I can see challenges in that as well’. (Participant).

### 3.6 Suggestions

Participants suggested that providers ask about their level of comfort using technology and offer increased support and resources for individuals with limited access to technology. Additionally, participants recommended providers determine steps to ensure they will have privacy during sessions, especially because many live with their families, and discuss expectations to be on‐ or off‐camera.

Moreover, participants brought up the need for flexibility in the standard hours of operation and noted the value of brief check‐ins with providers (e.g., via text or cellphone) suggesting more frequent meetings with the teams. While virtual platforms have improved, participants noted ease of use is critical. They suggested more groups in person to connect and socialize with others (e.g., going out to parks and activities), and noted the value of a hybrid model that offers options of in‐person and telehealth services.

## 4 DISCUSSION

This project examined the perspectives of young people and family members receiving OnTrackNY services about telehealth‐delivered treatment. The interviews and focus groups showed that overall, participants and family members expressed being comfortable with technology. Despite a learning curve using technology, individuals in this sample reported that they continued to receive a variety of services and meet with the team members of their choice, suggesting that the team‐based approach was preserved even as treatment shifted to telehealth. Challenges included connectivity, technical and accessibility issues. Participants also noted more difficulty forming social connections, particularly in group sessions and early on in treatment while establishing trust and rapport with the team, favouring a hybrid approach.

Participant and family perspectives were different with respect to the engagement in OnTrackNY services using telehealth. Most participants focused on positive aspects, such as feeling comfortable receiving OnTrackNY services via telehealth, feeling less judged, more able to fully be themselves or experiencing less paranoia leading up to remote appointments compared to in‐person. Furthermore, participants appreciated the ability to ‘*tune out*’ and have the camera on or off, or to have some flexibility during sessions that in‐person interactions do not allow. In contrast, some families were more likely to express concerns about their loved one's engagement in telehealth sessions and their ability to concentrate or actively participate. Similarly, when issues such as the presence of others during telehealth sessions arose, participants noted privacy concerns while family members mentioned the benefits of being more aware of their loved one's interactions with their team. While families were asked about their own experiences receiving care in OnTrackNY and their experiences with the care of their loved ones, they talked more about the experiences with the care of their loved ones.

Previous research findings indicate that individuals with early psychosis are generally accepting, proficient and willing to use technology as part of their care (Lal et al., 2022; Hoffman et al., 2020). Our findings reveal that most challenges were related to connectivity and technological issues, as well as a diminished sense of connectedness, aligning with existing literature (Randall et al., 2022). The results of our project show that participants and families continued to receive a variety of services via telehealth, suggesting that maintaining the team‐based approach in early psychosis services is feasible and that telehealth services are well‐received by young people (Dong et al., 2023; Lal et al., 2022; Nelson et al., 2022).

While participants in this project mostly reported positive experiences with telehealth, family members expressed concerns about their loved ones' engagement in sessions. Privacy concerns in the home and cybersecurity issues appeared in our sample, especially regarding the presence of others at home during telehealth visits. This contrasts with other studies where more than 80% of the sample did not report any confidentiality concerns (Lal et al., 2022). Although participants in this study expressed a preference for in‐person groups, one study reported high adherence and satisfaction with telehealth‐based psychotherapy groups for early psychosis (Wood et al., 2021). Future research may elucidate strategies to enhance sense of connectedness in group settings.

This quality improvement project described some of the benefits, challenges and experiences of young adults and their families with telehealth. The OnTrackNY program utilized information gleaned from this project to shape staff training (e.g., recommendations regarding in‐person engagement early in treatment), and disseminated findings to all stakeholders through accessible reports summarizing key takeaways. As early psychosis programs continue to offer telehealth options to their participants, it is likely beneficial to discuss the details of how and where telehealth will be used with the young person and to adopt a proactive problem‐solving approach to emerging challenges. Programs should consider offering a hybrid option and keep in mind that social connectedness is an important element of building rapport and trust.

Limitations of our report include findings not being representative of the full spectrum of participants and families' experiences. Particularly, all interviews and focus groups were conducted remotely and in English, likely missing the experiences of individuals with no or limited access to technology and who are most comfortable in other languages. The project sample was not representative of the entire OnTrackNY network, which spans all of New York State. The input of minors was absent in this sample and the average educational level was higher than the average of all OnTrackNY participants. Nevertheless, to our knowledge, this is the first project to qualitatively examine the experiences of participants and family members with telehealth early psychosis services and to elucidate some of the challenges and perceived advantages of receiving care virtually, with the additional strength of involving individuals with lived experience in every phase of the project.

## 5 CONCLUSIONS

This quality improvement project reported on the experiences of OnTrackNY participants and family members with the use of telehealth during the COVID‐19 pandemic. While the use of technology was generally well received, mixed perspectives from participants and family members on engagement during sessions and therapeutic alliance warrant further research. Findings highlight the importance of rapport building and engagement. Future work should focus on the quality of virtual interactions, engagement and impact of telehealth on treatment outcomes.

## FUNDING INFORMATION

This work was supported by the National Institute of Mental Health at the National Institutes of Health (R01MH120597 and R01MH120597‐02S1).

## Data Availability

Participants of this study did not provide consent for their data to be shared publicly and supporting data are not available.
